# Eosinophilic Granulomatosis With Polyangiitis Presenting With Rare Neurological Manifestations

**DOI:** 10.1155/crnm/5515152

**Published:** 2026-09-15

**Authors:** Valérie Gagnon Bruneau, Anne-Marie Lavigne

**Affiliations:** ^1^ Internal Medicine Department of Internal Medicine, Université Laval, Quebec, Canada, ulaval.ca; ^2^ Neurology Department of Neurology, Université Laval, Quebec, Canada, ulaval.ca

**Keywords:** ANCA-associated vasculitis, Churg–Strauss syndrome, eosinophilia, eosinophilic granulomatosis with polyangiitis (EGPA), myopathy, peripheral neuropathy, polyneuropathy

## Abstract

Eosinophilic granulomatosis with polyangiitis (EGPA) is a rare disease characterized by inflammation of small and medium‐sized blood vessels, leading to damage in various organs. It is classically associated with asthma, eosinophilia, and multisystem involvement, including frequent peripheral nervous system manifestations. We report the case of a 72‐year‐old woman with a history of asthma, rhinitis, and strokes, who presented with progressive muscle weakness, myalgia, and distal sensory deficits. Neurological examination revealed proximal muscle weakness and a length‐dependent sensory loss. Laboratory investigations demonstrated marked eosinophilia, elevated creatine kinase, and positive antimyeloperoxidase antibodies with an atypical cytoplasmic ANCA pattern. Imaging showed diffuse muscular hypermetabolism on positron emission tomography, while electromyography and nerve conduction studies revealed a length‐dependent axonal polyneuropathy with mild myopathic features. Muscle biopsy demonstrated mild nonspecific myopathic changes without definite evidence of vasculitis or eosinophilic infiltrates. Based on the 2022 American College of Rheumatology/European Alliance of Associations for Rheumatology classification criteria, a diagnosis of EGPA with neurological involvement was established. The patient was treated with high‐dose corticosteroids and rituximab, resulting in significant clinical and biochemical improvement at 3 months. This case illustrates the coexistence of atypical neurological manifestations of EGPA, including prominent myopathy, length‐dependent polyneuropathy, and retrospectively suspected central nervous system vasculitic involvement, emphasizing the importance of early recognition and multidisciplinary management.

## 1. Introduction

Formerly known as Churg–Strauss syndrome, eosinophilic granulomatosis with polyangiitis (EGPA) is a systemic small‐ to medium‐vessel vasculitis that belongs to the spectrum of antineutrophil cytoplasmic antibody (ANCA)‐associated vasculitides [1]. Unlike GPA and microscopic polyangiitis (MPA), EGPA is characterized by marked eosinophilic inflammation and a strong association with late‐onset asthma and allergic manifestations [2]. ANCA is detected in only 30%–40% of patients with EGPA [3], a lower positivity rate than that observed in GPA and MPA. In addition, prominent eosinophilic tissue infiltration and the clinical heterogeneity of EGPA support the contribution of both ANCA‐mediated vasculitic mechanisms and eosinophil‐driven inflammation to tissue injury [4]. Neurological involvement is common, particularly during the vasculitic phase of the disease. Peripheral nervous system (PNS) involvement occurs in more than half of patients and typically presents with mononeuritis multiplex [5], whereas central nervous system (CNS) involvement is much less common, occurring in approximately 4%–5% of cases [6, 7]. However, atypical presentations such as myopathy are less frequently described and may complicate the diagnostic process.

## 2. Case Presentation

A 72‐year‐old woman was admitted with generalized muscle weakness and myalgia. Her medical history was notable for asthma, rhinitis, nasal polyposis, and dyslipidemia. She had been evaluated 2 years earlier in rheumatology for eosinophilia, which resolved spontaneously, and 1 year prior in neurology for multiple lacunar ischemic lesions in the bilateral centrum semiovale.

Manual muscle testing using the Medical Research Council scale showed symmetrical proximal muscle weakness. In the upper limbs, shoulder abduction was 4+/5, while elbow flexion and extension, wrist flexion and extension, and finger abduction were preserved. In the lower limbs, hip flexion and knee flexion were 3/5, knee extension was 4/5, and ankle dorsiflexion and plantarflexion were 5/5 symmetrically. Reflexes were brisk in the arms, with a bilateral Hoffman sign. In the lower limbs, patellar reflexes were preserved, whereas Achilles reflexes were absent. Sensory examination revealed a length‐dependent loss of pinprick and temperature sensation in a stocking‐and‐glove distribution, with preserved vibration sense.

Laboratory investigations revealed marked eosinophilia, elevated creatine kinase, increased C‐reactive protein, and erythrocyte sedimentation rate, along with positive C‐ANCA and antimyeloperoxidase (MPO) antibodies (Table 1). The autoimmune panel, genetic, and infectious workups were unremarkable.

**TABLE 1:** Main blood test results.

Tests	Results	Normal ranges
Leukocytes	18 × 10^9^/L	4.20–10.5 10^9^/L
Absolute eosinophil count	11.4 × 10^9^/L	< 0.5 × 10^9^/L
Hemoglobin	120–125 g/L	115–155 g/L
Platelets	601 × 10^9^/L	150–400 × 10^9^/L
Creatine kinase	5832 U/L	30–135 U/L
C‐Reactive protein (CRP)	111.7 mg/L	< 10 mg/L
Erythrocyte sedimentation rate (ESR)	40 mm/h	< 20 mm/h
Creatinine clearance	40–50 μmol/L	45–85 μmol/L
Vitamin B12	1324 pmol/L	> 135 pmol/L
Tryptase	7.1 μg/L	< 11.4 μg/L
Antinuclear antibodies (ANA)	1: 320 (speckled)	—
C‐ANCA	1: 640	—
P‐ANCA	Negative	—
Anti‐MPO	> 134 Ul/mL	< 3.5 Ul/mL
Anti‐PR3	< 0.2 UI/mL	< 2 UI/mL
IgG4	5.12 g/L	0.4–0.86 g/L
IgE	1360 kUI/L	< 100 kUI/L
Red blood cells in urine	25/high power fields	< 3/high power fields

Abbreviations: ANA, antinuclear antibodies; C‐ANCA, cytoplasmic antineutrophil cytoplasmic antibodies; CK, creatine kinase; CRP, C‐reactive protein; ESR, erythrocyte sedimentation rate; IgE, immunoglobulin E; IgG4, immunoglobulin G subclass 4; MPO, myeloperoxidase; P‐ANCA, perinuclear antineutrophil cytoplasmic antibodies; PR3, proteinase 3.

**FIGURE 1:**
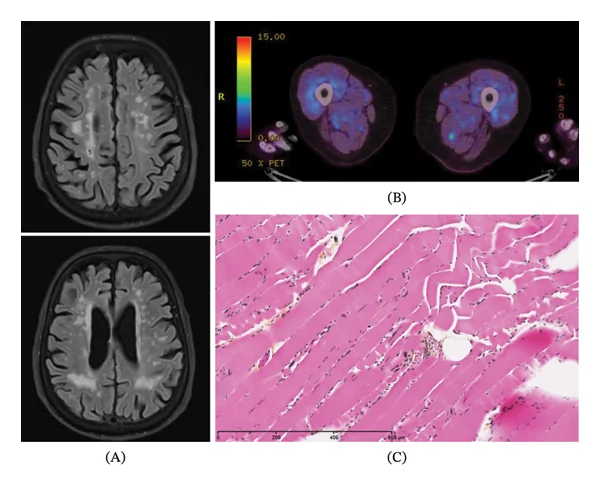
Radiological and histopathological findings. (A) Axial FLAIR brain magnetic resonance imaging showing chronic lacunes and a moderate leukoencephalopathy. (B) Positron emission tomography demonstrating diffuse and heterogeneous muscular hypermetabolism in both thighs. (C) Hematoxylin and eosin staining of thigh muscle biopsy demonstrating mild nonspecific myopathic changes with mild fiber size variability and rare atrophic fibers, without evidence of vasculitis or significant inflammatory or eosinophilic infiltrates.

Positron emission tomography demonstrated diffuse and heterogeneous muscular hypermetabolism. Computed tomography angiography of the chest and abdominopelvic regions showed no evidence of vasculitis or ground‐glass opacities. Transthoracic echocardiography revealed no cardiac involvement. Brain and cervical magnetic resonance imaging showed supratentorial chronic ischemic sequelae without evidence of recent ischemia, hemorrhage, or myelitis. Electromyography and nerve conduction studies demonstrated a mild axonal polyneuropathy with myopathic features. Muscle biopsy from the thigh demonstrated mild nonspecific myopathic changes without evidence of vasculitis or eosinophilic infiltrates (Figure 1).

Atorvastatin was discontinued, and rhabdomyolysis was managed with intravenous hydration. Treatment included 5 days of intravenous methylprednisolone followed by an oral prednisone taper. Rituximab 1g was administered during the hospitalization, with a second dose given 2 weeks later.

The diagnosis of EGPA with myositis was based on the 2022 ACR/EULAR classification criteria (Table 2) [8], with a total score of 8 points (threshold ≥ 6 for diagnosis).

**TABLE 2:** 2022 ACR/EULAR classification criteria for EGPA.

Criteria	Results	Our patient
*Clinical criteria*		
Obstructive airway disease	+3	+3
Nasal polyps	+3	+3
Mononeuritis multiplex	+1	+1

*Laboratory and biopsy criteria*		
Blood eosinophil count ≥ 1 × 10^9^/L	+5	+5
Extravascular eosinophilic‐predominant inflammation on biopsy	+2	
Positive test for C‐ANCA or anti‐PR3	−3	−3
Hematuria	−1	−1

Total		8

Abbreviations: C‐ANCA, cytoplasmic antineutrophil cytoplasmic antibodies; PR3, proteinase 3.

At the 3‐month follow‐up, the patient demonstrated marked clinical improvement in her strength and sensitive symptoms while remaining on prednisone 20 mg daily. Laboratory parameters normalized, including creatine kinase, eosinophil count, and inflammatory markers. Repeat electromyography with nerve conduction studies 2 months later showed mild progression of axonal polyneuropathy without active denervation or myopathic changes, interpreted as delayed electrophysiological lag. Prednisone was gradually tapered and completely discontinued. At the 22 month follow‐up, the patient had been treated exclusively with rituximab for the previous 18 months. She continued to demonstrate complete clinical and biochemical remission.

## 3. Discussion

This case illustrates an atypical neurological presentation of EGPA characterized by prominent myopathy, symmetric length‐dependent polyneuropathy, and lacunar ischemic lesions. Neurological manifestations differ across ANCA‐associated vasculitides. In GPA, granulomatous inflammation more commonly involves the CNS, often manifesting as hypertrophic pachymeningitis and cranial neuropathies. In contrast, EGPA predominantly affects the PNS, classically presenting as mononeuritis multiplex [5, 10]. However, approximately 10% of patients with EGPA may present with a symmetric length‐dependent polyneuropathy [9], as observed in our patient.

Myalgias are reported in 37%–57% of patients with EGPA [11], yet true myositis with objective weakness and markedly elevated creatine kinase is rare. Only a limited number of cases describe myopathy as a primary manifestation [11–15], and histopathological confirmation is often lacking. Although necrotizing vasculitis with eosinophilic infiltration has been reported in muscle biopsies, findings may be nonspecific, as in our case. The absence of eosinophilic infiltration on muscle biopsy may reflect sampling limitations or the patchy nature of EGPA involvement. It also highlights the importance of a clinical approach integrating clinical presentation, laboratory abnormalities, and classification criteria, rather than relying solely on tissue confirmation.

Although no active CNS involvement was identified at presentation, the extent of chronic lacunar infarcts and leukoencephalopathy on neuroimaging raised the possibility of prior CNS vasculitic involvement that may have preceded the diagnosis of EGPA. While CNS involvement is uncommon, occurring in approximately 4%–5% of cases [6, 7], its recognition is critical given the potential for significant morbidity. Reported manifestations are heterogeneous and include ischemic stroke, intracerebral hemorrhage, visual loss (including optic neuritis, central retinal artery occlusion, and cortical blindness), as well as cranial neuropathies [16]. Ischemic cerebrovascular lesions appear to be the most frequently reported CNS manifestation [16]. The coexistence of peripheral and CNS manifestations also supports a systemic vasculitic process and reinforces the need for comprehensive neurological evaluation in atypical presentations.

The patient’s serological profile further is also suggestive of a vasculitic phenotype. EGPA is classically associated with anti‐MPO antibodies, typically producing a perinuclear ANCA pattern, although atypical cytoplasmic patterns may occur in approximately 5% of cases [3]. ANCA positivity has been associated with a vasculitic phenotype, particularly involving the PNS through small‐vessel involvement of the vasa nervorum. In contrast, ANCA‐negative disease is more often characterized by eosinophil‐driven tissue infiltration. Although this distinction is not absolute, it provides a useful framework for understanding disease heterogeneity [1, 17].

The severity and rapid progression of neurological involvement guided therapeutic decisions. While immunotherapy in ANCA‐associated vasculitis focuses on remission induction and maintenance, there are no established treatments specifically targeting myopathy or polyneuropathy in EGPA and high‐quality evidence remains limited. Current recommendations are largely based on expert consensus [18]. Severe disease warrants induction with high‐dose glucocorticoids in combination with rituximab or cyclophosphamide. Rituximab is generally favored in ANCA‐positive disease, whereas cyclophosphamide may be considered in ANCA‐negative patients or in cases with cardiac or gastrointestinal involvement [18].

In this case, the patient’s rapidly progressive weakness with functional impairment supported classification as severe disease. Although muscle involvement is not included in standard severity scores, its clinical impact justified early escalation of immunosuppressive therapy. The favorable clinical and biochemical response to corticosteroids and rituximab further supports the effectiveness of this approach in neurologically predominant EGPA.

Overall, this case underscores the diagnostic and therapeutic challenges posed by atypical neurological presentations of EGPA. It emphasizes that EGPA should remain in the differential diagnosis of patients presenting with eosinophilia and unexplained neuromuscular manifestations, including myopathy and peripheral neuropathy. Early recognition and timely immunosuppressive treatment are essential for favorable neurological outcomes. Further studies are required to characterize further rare manifestations such as EGPA‐associated myopathy and to inform evidence‐based management strategies.

## Disclosure

The authors used a large language model to assist with language editing and manuscript refinement. All content was reviewed, revised, and approved by the authors, who take full responsibility for the final manuscript.

## Ethics Statement

We confirm that we have read the Journal’s position on issues involved in ethical publication and affirm that this report is consistent with those guidelines.

## Consent

Written informed consent was obtained from the patient for publication of this case report and accompanying images.

## Conflicts of Interest

The authors declare no conflicts of interest.
